# Collective dynamics of identical phase oscillators with high-order coupling

**DOI:** 10.1038/srep31133

**Published:** 2016-08-05

**Authors:** Can Xu, Hairong Xiang, Jian Gao, Zhigang Zheng

**Affiliations:** 1 Institute of Systems Science and College of Information Science and Engineering, Huaqiao University, Xiamen 361021, China; 2 Department of Physics, Beijing Normal University, Beijing 100875, China; 3 Beijing-Hong Kong-Singapore Joint Center for Nonlinear and Complex Systems (Beijing), Beijing Normal University, Beijing 100875, China

## Abstract

In this paper, we propose a framework to investigate the collective dynamics in ensembles of globally coupled phase oscillators when higher-order modes dominate the coupling. The spatiotemporal properties of the attractors in various regions of parameter space are analyzed. Furthermore, a detailed linear stability analysis proves that the stationary symmetric distribution is only neutrally stable in the marginal regime which stems from the generalized time-reversal symmetry. Moreover, the critical parameters of the transition among various regimes are determined analytically by both the Ott-Antonsen method and linear stability analysis, the transient dynamics are further revealed in terms of the characteristic curves method. Finally, for the more general initial condition the symmetric dynamics could be reduced to a rigorous three-dimensional manifold which shows that the neutrally stable chaos could also occur in this model for particular parameters. Our theoretical analysis and numerical results are consistent with each other, which can help us understand the dynamical properties in general systems with higher-order harmonics couplings.

Synchronization of coupled oscillators is an important issue in a wide variety of areas throughout the nature, and it has attracted much attention from the scientific community during the last decades^1^. Examples include the flashing of fireflies^2^, electrochemical and spin-toque oscillators^3^,^4^, pedestrians on footbridges^5^, applauding person in a large audience^6^ and others. Understanding the intrinsic dynamical properties of such system is therefore of considerable theoretical and experimental interest. Indeed, in the weak interaction limit, the dynamics of limit-cycle oscillators could be effectively described in terms of their phase variable *θ*. While the most famous case is the Kuramoto model^7^, it stands out as the classical paradigm for studying the spontaneous emergence of collective synchronization in such system^8^. The form of phase equation obeys,


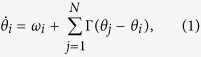


where *θ*_*i*_ denotes the phase of the *i*th oscillator, *ω*_*i*_ is its natural frequency. Γ(*θ*) is 2*π*-periodic function representing the interaction between units. The simple choice of Γ(*θ*) = (*K*/*N*) sin *θ* leads to the classical Kuramoto model. Along the past decades the Kuramoto model with its generalizations have inspired and simulated a wealth of extensive studies because of both their simplicity for mathematical treatment and their relevance to practice^9^,^10^,^11^. In particular, when Γ(*θ*) includes higher harmonics, the system exhibits nontrivial dynamical features which have been reported in the recent works^12^,^13^,^14^,^15^.

In many realistic systems, the higher-order harmonic term (especially the second) often plays an essential role in the interaction and even dominates the coupling function. Such as the Huygens pendulum system^16^, the neuronal oscillators and genetic networks system^17^, the globally coupled photochemical oscillators system^18^, etc. In contrast to the pervious discussions of Kuramoto model containing higher order coupling, in the present work, we study phase equations of the following form,


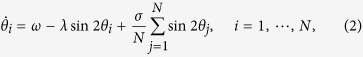


where *ω* is the frequency of identical oscillators, *λ* and *σ* are the global coupling strengths respectively. There are various motivations for the study of the current model. For example, in the Josephson junction arrays^19^,^20^, the dynamics of a single element is determined by a double well potential and therefore the strong effects caused by the second harmonics is important. Another example is the star-like model with a central element and *N* leaves, where the case of single harmonic coupling was considered in the paper^21^,^22^,^23^,^24^,^25^,^26^,^27^,^28^,^29^. Similar to the mean-field coupling in the Kuramoto model, the interaction term in Eq. (2) turns out to be a driving force that does not depend on the phase of driven unit and is equally on every oscillator.

In this paper, we present a complete framework to investigate the collective dynamics of globally coupled phase oscillators when higher-order modes dominate the coupling function. It includes several aspects. First, we use the Ott-Antonsen method^30^ to obtain the low-dimensional description of symmetric dynamics, and various states, such as the double-center, single-center, center-synchrony coexistence, and synchrony regimes, respectively are illustrated schematically in the phase diagram (Fig. 1). Furthermore, a detailed linear stability analysis based on the self-consistent approach is implemented and both the boundary curves between various steady states and eigenvalues of then are obtained analytically, which are consistent with the Ott-Antonsen method. Additionally, it has been proved that the linearized operator for the stationary symmetric distribution has infinitely many purely imaginary eigenvalues, which implies the stationary symmetric distribution is neutrally stable to perturbations in all the directions. Second, the two-cluster synchrony state is demonstrated which is initial-value dependent, and the general transient solutions of the distribution are calculated in term of the characteristics method. Finally, the general initial conditions for the phase oscillators which lie off the Poisson submanifold are considered, where the symmetric dynamics are governed by the three-dimensional Möbius transformation rigorously. Consequently, one can expect the chaotic behavior of both the symmetric dynamics *r*_2_ and the degree of asymmetry *r*_1_ to occur in the marginal regime. Extensive numerical simulations have been carried out to verify our theoretical analysis, in the following we report our main results both theoretically and numerically. These different aspects together present a global picture for the understanding of the dynamical properties in this system.

## Results

### Symmetric dynamics

We start by considering the high-order coupled phase oscillators model (2), without loss of generality, the range of the coupling strength is restricted to *λ* > 0 and −∞ < *σ* < +∞ throughout the paper. The most important characteristic of the current model is the introduction of higher harmonics in the coupling function, and hence the generalized order parameters characterizing the collective behavior of the system^12^ are needed, which yield,


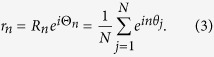


For *n* ∈ integer, *R*_*n*_ is the magnitude of the complex, *n*-th order parameter, and Θ_*n*_ is its phase. As named in ref. 12, the amplitude *R*_2_ measures the level of cluster synchrony, while *R*_1_ measures the degree of asymmetry in clustering. Eq. (2) can be rewritten in terms of *r*_2_ as


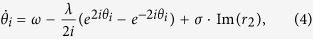


where Im represents the imaginary part. In the thermodynamic limit *N* → ∞, Eq. (4) is equivalent to the continuity equation as a consequence of the conservation of the number of oscillators, i.e.,


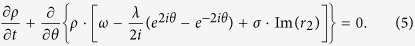


Here *ρ*(*θ*, *t*)*dθ* gives the fraction of oscillators which lie between *θ* and *θ* + *dθ* at time *t* with the appropriate normalization condition 
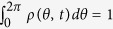
. On its turn, the continuity limit of the generalized order parameters takes the form


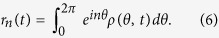


Additionally, considering the 2*π*-periodicity of *θ* in the distribution function *ρ*(*θ*, *t*), which allows a Fourier expansion and can be written as


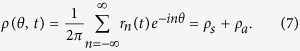


It is obvious that the *n*-th Fourier coefficient of *ρ*(*θ*, *t*) is just the *n*-th order parameter Eq. (6). Here *ρ*_*s*_ is the sum where *n* is even and symmetric in the sense that *ρ*_*s*_(*θ* + *π*, *t*) = *ρ*_*s*_(*θ*, *t*), and *ρ*_*a*_ is the sum where *n* is odd which is antisymmetric with respect to the translation by *π*, *ρ*_*a*_(*θ* + *π*, *t*) = −*ρ*_*a*_(*θ*, *t*). Then, substituting the expansion Eq. (7) into Eq. (5), we obtain two sets of equations as,


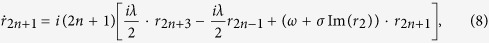


for the odd Fourier coefficients, and


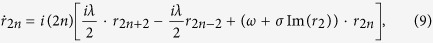


for the even Fourier coefficients. Eq. (8) together with Eq. (9) provide two sets of infinitely many coupled equations which evolve independently, for instance, the motion of *r*_2_ is not only dependent on itself but also governed by *r*_4_ and *r*_0_. Going further, observing the specific form of Eqs (8) and (9), one notices that Eq. (8) has a trivial invariant manifold solution


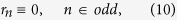


and Eq. (9) has a non-trivial invariant manifold solution


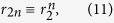


which is indeed the Ott-Antonsen ansatz^30^,^31^. The Ott-Antonsen method yields a special solution for the system provided that *r*_2_ evolves according to a single ordinary differential equation(ODE) called the Riccati equation,


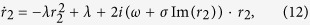


and the solution of this kind turns out to form a two-dimensional invariant manifold, which is the set of Poisson kernels


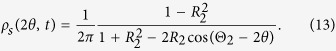


One central issue regarding the study is to identify all the possible collective states either stationary or nonstationary, and reveal various bifurcations as the change of the coupling parameter *λ* and *σ*, provided that the initial symmetric distribution has the form of Poisson kernels.

The Riccati equation (12) describes the collective symmetric dynamics of Eq. (2) in terms of the second order parameter *r*_2_, and it can be straightforwardly rewritten in cartesian coordinates *r*_2_ = *x* + *iy* as


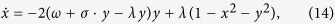



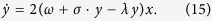


In the phase space of the second-order parameter, the natural boundary is *x*^2^ + *y*^2^ = 1, a fixed point is determined by the intersection of nullclines 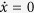 and 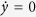 within the boundary. One recalls that the first point is *y*_01_ = *ω*/(*λ* − *σ*) and 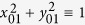, which is on the unit circle and defined as the synchrony state, and the synchrony state exists when |*λ* − *σ*| ≥ *ω*. Additionally, the linearization Jacobian matrix of the synchrony state has two eigenvalues *δ*_1_ = −2*λx*_01_ and *δ*_2_ = −2(*λ* − *σ*)*x*_01_, which determines the stability of the synchrony state in the existence regimes. Once again, the same strategy such as the existence and the stability analysis can be actually adopted for the second fixed point *x*_02_ = 0, 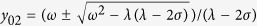, which is on the *y* axis and termed as the splay state^32^. The Jacobian matrix of the second point has two purely imaginary eigenvalues, which shows that the splay state is neutrally stable to perturbations and is a center in the Ott-Antonsen manifold.

**Figure 1:**
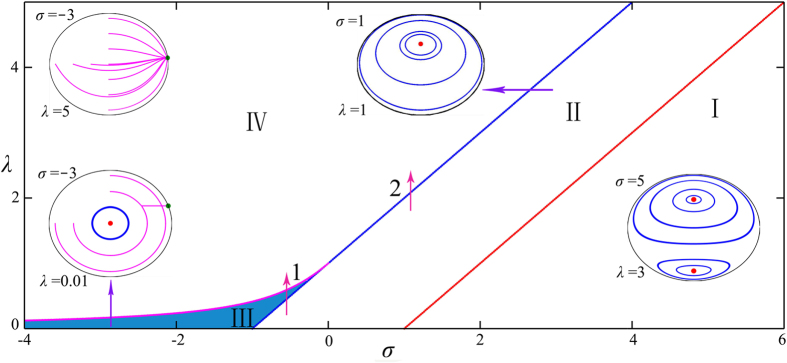
Phase diagram of the symmetric dynamics in the parameter space *σ* and *λ*. I is the double center regime, II is the single center regime, III is the center-synchrony coexistence regime, IV is the synchrony regime, respectively. The boundary curves are *λ* = *σ* − *ω* from I to II, *λ* = *σ* + *ω* from II to III or IV, 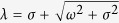 from III to IV, in the numerical simulations we set *ω* = 1.

Figure 1 presents the phase diagram that summarizes the results of our analysis of Eqs (14) and (15), and we find that there are four different dynamical regimes, i.e., the double-center regime I, the single-center regime II, the center-synchrony coexistence regime III and the synchrony regime IV, respectively. In addition, various bifurcations and transitions among these states are illustrated when the parameters are adjusted to pass through the boundary curves. There are two kinds of routes to synchrony, for instance, when we start from II to IV (arrow 1 in the Fig. 1), the transition to synchrony is a first-order phase transition with hysteresis because of the coexistence regime III. However, when we turn to the second route (arrow 2 in the Fig. 1) the transition is discontinuous and is absent of hysteresis.

The above analysis reveals the low-dimensional collective behaviors of symmetric dynamics, where the bifurcations and transitions among various states are presented. Actually, all the results are within the framework of the Ott-Antonsen invariant manifold including the linear perturbation of the fixed points. In the following, we perform a thorough linear stability analysis of the stationary symmetric distribution in terms of the self-consistent theory^33^, where all the boundary curves could be obtained analytically in an alternative way. As it will appear momentarily, when we impose a general perturbation for the steady states, the analytical predictions above are still valid. Meanwhile, the eigenvalues of the steady states keep the same form above and the splay state turns out to be neutrally stable in all directions.

A convenient way of solving the symmetric dynamics is to make the change of variable *φ* = 2*θ*, which yields a new dynamical equation


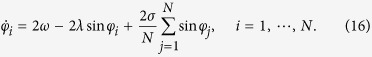


Eq. (16) has the form of dynamical equations of over damped linear Josephson junction arrays. It is obvious that the distribution of *φ* is equivalent to the *ρ*_*s*_ in Eq. (13). The synchrony state corresponds to a spatially homogeneous fixed point of Eq. (16) defined as 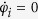, for all the *i* and 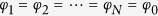 which is the simplest attractor. Therefore, this phase leads to a solution of equation


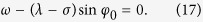


In this case sin *φ*_0_ = *ω*/(*λ* − *σ*), and the Jacobian matrix of Eq. (16) is a circulant matrix^23^,^33^ with two kinds of eigenvalues. The first one is


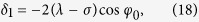


which corresponds to a spatial homogeneous fluctuation, and the other *N* − 1 eigenvalues are


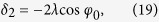


which have (*N* − 1)-fold degeneracy that correspond to inhomogeneous fluctuations. Incidentally, one notes that *x*_01_ ≡ cos *φ*_0_, *y*_01_ ≡ sin *φ*_0_, this result is consistent with a basic fact that the synchrony state is contained in the Ott-Antonsen manifold. Numerical experiment shows that the basin of attraction of the synchrony state in regime IV is global.

Another scenario is the stationary distribution where the phase *φ* is smoothly distributed over [0, 2*π*], and from Eq. (16) one observes that the general form of such a stationary distribution is


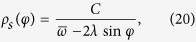


where 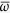 is the effective frequency, which can be determined self-consistently from the equation


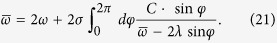


*C* is the normalization constant as 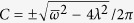, when 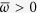, *C* is positive and vice versa. Substituting the expression of *C* into Eq. (21), we obtain the solution of 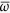,


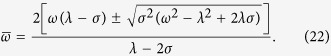


It should be emphasized that the stationary distribution *ρ*_*s*_(*φ*) corresponds to the splay state in the invariant manifold above, because the distribution Eq. (20) has the form of Possion kernels, and


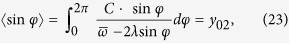


and


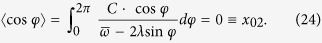


The perturbation of the continuity equation for the stationary symmetric distribution *ρ*_*s*_(*φ*) is


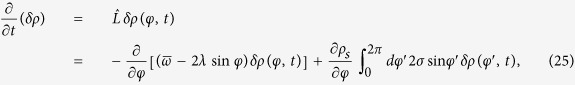


and the eigenvalue equation (25) is convenient to be treated in the function space,


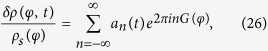


where *G*(*φ*) is the basis function


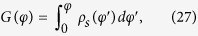


and *a*_*n*_(*t*) are the expansion coefficients. The stability analysis of the stationary distribution (see in the Supplementary Material) indicates that in regimes I, II and III, all the infinitely many eigenvalues of the operator *L* are purely imaginary, which implies that the stationary distribution is neutrally stable in all directions, not only confined to the Ott-Antonsen manifold. The evidence of a regime where the stationary distribution is marginally stable is termed as the marginal regime^33^ and in which there are no particular attractors that the system converges to. Actually, this highly non-generic property is associated with the time-reversal symmetry that the Eq. (16) exhibits. When we start in the Ott-Antonsen manifold, the trajectory of *r*_2_ is a two-dimensional closed periodic curve (see the insert of Fig. 1), however, when we start with a more general initial condition in the marginal regime, the situation differs substantially as we will see in the following part.

### The steady and transient dynamics

In the above analysis, we investigated the symmetric dynamics by using two kinds of ways, both the Ott-Antonsen method and the self-consistency theory. One recalls that when the parameters are in regime IV, the symmetric dynamics converge to the steady state, as a result, the two-synchrony-cluster state emerges, i.e., sin 2*θ*_0_ = *ω*/(*λ* − *σ*), cos 2*θ*_0_ > 0. Thus, the complete steady state distribution of phase oscillators is


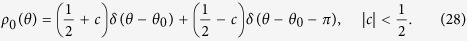


**Figure 2:**
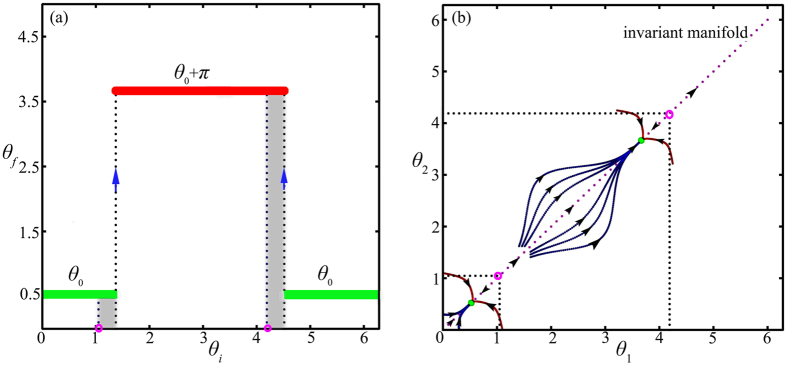
(**a**) The initial phase *θ*_*i*_ vs the final phase *θ*_*f*_ when we choose the initial phase distribution *ρ*(*θ*, *t*_0_) = (1 + cos *θ*)/2*π* and the number of oscillator is *N* = 10000 in the simulation, when the initial phase is in the gray area, these oscillators will bypass the saddle points (the pink hollow circle in the horizontal axis) which leads to *ε* ≠ 0. (**b**) The diagram of the Arnold diffusion mechanism in the same parameter with *N* = 3 in terms of the phase space of *θ*_1_ and *θ*_2_. For some particular trajectory, such as the red line, the oscillator will bypass the saddle point in the invariant manifold.

*ρ*_0_(*θ*) is comprised of two delta functions denoting two clusters of oscillators at *θ*_0_ = 0.5 arcsin *ω*/(*λ* − *σ*) and *θ*_0_ + *π*. Correspondingly, the phase oscillators settle to one of the two stable equilibria, while the unstable equilibria *π*/2 − *θ*_0_ and 3*π*/2 − *θ*_0_ serve as the boundaries for the basin of attraction. Therefore, the degree of asymmetry |*r*_1_| is


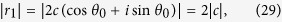


which depends on the free parameter *c* and could be determined approximatively from initial condition. Note that 1/2 − *c* is just the fraction of oscillators in the locked phase *θ*_0_ + *π*, namely,


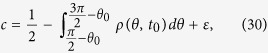


where *ρ*(*θ*, *t*_0_) is the initial density, *ε* is the error caused by the Arnold diffusion. Theoretically, when the initial phase is in the one-dimensional invariant manifold 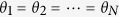 the evolution of phase *θ*(*t*) for all oscillators can never pass through two saddle points *π*/2 − *θ*_0_ and 3*π*/2 − *θ*_0_ which means *ϵ* = 0. However, for the more general initial conditions, some oscillators that are in the neighborhood of two unstable equilibrium states can bypass the saddle points (Fig. 2(b) illustrates schematically the mechanism in the low-dimensional phase space where *N* = 3) and therefore *ε* is non-negligible. Figure 2(a) presents the numerical simulation when we choose *θ*_0_ = *π*/6 and *ρ*(*θ*, *t*_0_) = (1 + cos *θ*)/2*π*, it is clear that the initial phases in the gray area eventually bypass the saddle points (the pink hollow circle in the horizontal axis). This interesting phenomenon can be heuristically understood as follows, those oscillators in the gray area tend to choose a relatively near equilibrium state to settle in the high- dimensional phase space, while this is forbidden in the one-dimensional invariant manifold.

When the symmetric dynamics *r*_2_ approaches the steady state, then the |*r*_1_| dynamics reaches the steady state quickly. However, in a large marginal regime of the phase diagram Fig. 1, the dynamics of *r*_2_ can never converge to an attractor, and the motion of *r*_2_ is time-dependent. To capture the dynamical features of *r*_1_, we can solve the partial differential equation (PDE) (5)


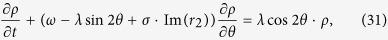


in terms of the characteristics method. When we start in the characteristic curve *θ*(*t*, *t*_0_), the distribution function will be *ρ*(*θ*, *t*) ≡ *ρ*(*θ*(*t*, *t*_0_), *t*) and the PDE becomes the ODEs. The characteristic equations are


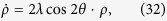



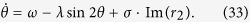


When the symmetric dynamics are in the Ott-Antonsen invariant manifold, the motion of *r*_2_ is governed by Eq. (12). Generally, Eqs (32) and (33) are difficult to solve analytically, while for some particular situations e.g., the orbit of *r*_2_ is near to the center point, the fluctuation of *r*_2_ is small enough reflecting that Im(*r*_2_) is approximately a constant. Then the expression for the characteristic curves *θ*(*t*, *t*_0_) starting at the initial phase *θ*_0_ yields


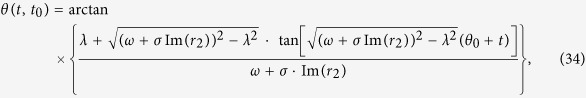


and the distribution along the characteristic equations is


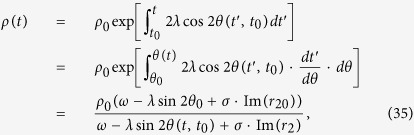


where *ρ*_0_ is the initial value of the distribution function, *r*_20_ is the initial value of *r*_2_. Theoretically, the general form of the density function *ρ*(*θ*, *t*) could be determined by substituting the inverse solution *θ*_0_(*θ*(*t*)) Eq. (34) into Eq. (35), and the generalized order parameters could also be obtained through the integral Eq. (6). Due to the multivalue feature of the anti-trigonometric function Eq. (34), it is convenient to calculate the first-order parameter *r*_1_ via the integral


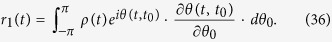


**Figure 3:**
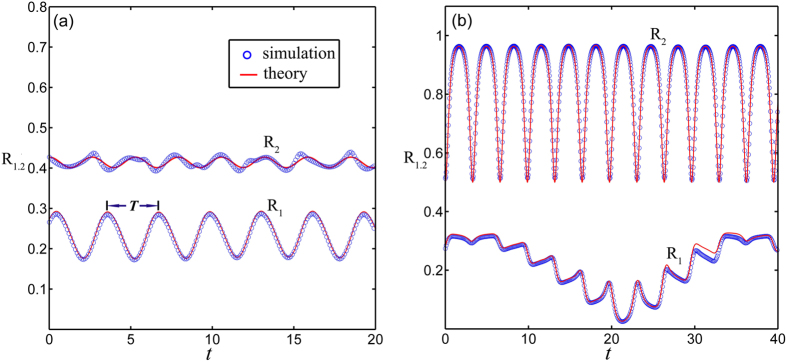
Transient dynamics of *R*_1_ and *R*_2_. (**a**) When the amplitude of *R*_2_ is small, *R*_1_ oscillates regularly with a period *T*, *σ* = 1.0, *λ* = 1.0, Re(*r*_20_) = 0, and Im(*r*_20_) = 0.427. (**b**) When the amplitude of *R*_2_ is large, *R*_1_ oscillates irregularly, *σ* = 1.0, *λ* = 1.4, Re(*r*_20_) = 0, and Im(*r*_20_) = −0.5. In the numerical simulation we choose the initial phase distribution *ρ*(*θ*, *t*_0_) = *ρ*_*s*_(2*θ*, *t*_0_) (1 + 0.5 cos(*θ*)) and *N* = 10000, the line is calculated by the characteristic theory and the circle is the numerical simulation.

From Eq. (36) and Fig. 3(a), we find that |*r*_1_(*t*)| oscillates regularly with a period


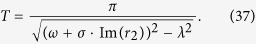


When the fluctuation of *r*_2_ is considerably large, the time dependence of *r*_1_ is irregular as shown in Fig. 3(b).

The discussion of the symmetric dynamics Eq. (16) exhibits a two-dimensional Ott-Antonsen manifold proving that the initial symmetric distribution takes the form of Possion kernels Eq. (13). In fact, the significant works point out that the evolution equations for the form of Eq. (16) are generated by the action of the Möbius group^34^,^35^


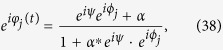


*α* is a complex variable, *ψ* is real, and *ϕ*_*j*_ is the motion constant. The group action partition the *N*-dimensional state space into a three-dimensional invariant manifold, and the three parameters Re(*α*), Im(*α*), *ψ* are governed by the following equations


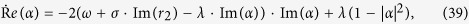



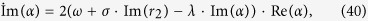



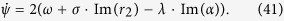


When the motion constant takes a general distribution, the parameter *r*_2_ can be written in terms of *α* and *ψ* as^34^


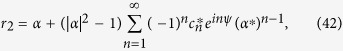


*c*_*n*_ is the *n*-th Fourier expansion coefficient of the distribution of motion constants. For the simple case, where the distribution is uniform on [0, 2*π*], *c*_*n*_ ≡ 0 for all the *n*, *r*_2_ ≡ *α*, and *α* decouples from *ψ*. This implies that the three-dimensional state space has a two-dimensional invariant submanifold which is indeed the Ott-Antonsen manifold. However, in the more typical case where *α* and *ψ* are interdependent, the three-dimensional equation can exhibit non-general dynamical features in the marginal regime.

**Figure 4:**
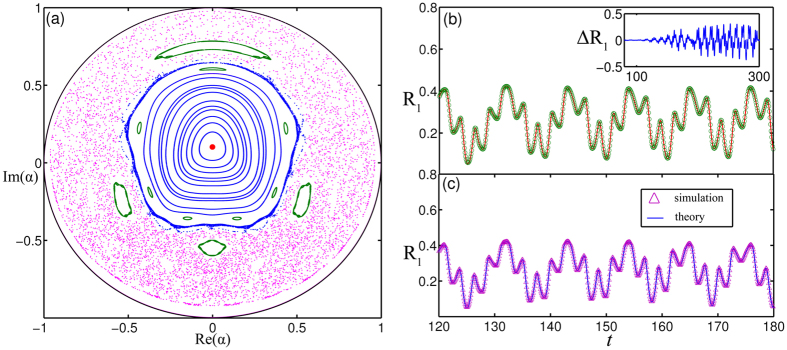
(**a**) Poincare section of Eqs (39)~(41) at *ψ*(mod 2*π*) = 0, quasiperiodic trajectories appear as closed curves or island chains, periodic trajectories appear as fixed points or period-*p* points of integer period, and chaotic trajectories fill the remaining region of the unit disk, where *ω* = 1.0, *λ* = 1.5, *σ* = 2.0. (**b**) *R*_1_ vs *t* in the chaos regime with *α*(0) = 0.5 + *i* 0.5 and *ψ*(0) = 0.0. (**c**) *R*_1_ vs *t* with adjacent parameter value *δα*(0) = 0.001, *δψ*(0) = 0. The insert is the difference of *R*_1_ vs *t* where we can see that the bias of the order parameter with neighboring parameters will be significant in the long time. In the simulation we choose *N* = 100000 and the initial phase distribution *ρ*(*θ*, *t*_0_) = *ρ*_*s*_(2*θ*, *t*_0_) (1 + 0.5 cos(*θ*)), where *ρ*_*s*_(2*θ*, *t*_0_) is determined by Eq. (38), the line is determined in terms of the the characteristic theory and the circle and triangle are calculated by the numerical simulation.

In Fig. 4(a), we use Poincare section at *ψ*(mod 2*π*) = 0 to sort out the structure of state space, where the parameters are chosen in regime II of Fig. 1 (*λ* = 1.5 and *σ* = 2.0), and the motion constant has a distribution (1 + sin *ϕ*)/2*π*. In the Poincare section, quasiperiodic trajectories appear as closed curves or island chains, periodic trajectories appear as fixed points or period-*p* points of integer period, and chaotic trajectories fill the remaining regimes of the unit disk. The picture of the phase portraits in the marginal regime resembles the phenomenon of Hamiltonian chaos in the classical integrable system, and the appearance of the quasi-Hamiltonian properties originates form the time reversibility symmetry under the transformation *t* → −*t*, *ψ* → −*ψ*, *x* → −*x*, that the evolution equations are invariant. When we choose the initial value in the chaos regime, *α*(0) = 0.5 + *i* 0.5, *ψ*(0) = 0.0, the three Lyapunov exponents are *λ*_1_ = −0.266, *λ*_2_ = 0, *λ*_3_ = 0.227, respectively. As a result, the evolution of order parameters *r*_2_ and *r*_1_ is also chaotic which are sensitive to initial values. Figure 4(b,c) depict the evolution of *R*_1_(*t*) with two adjacent parameter values where *δα*(0) = 0.001, *δψ*(0) = 0. It is clear that the bias of order parameter with neighboring parameters (the insert in the Fig. 4(b)) will be significant in the long time and the characteristic curve and numerical simulation are consistent with each other well.

## Discussion

To summarize, we investigated the collective dynamics of globally coupled identical phase oscillators when second harmonics dominate the coupling, and solutions can be decomposed into symmetric and antisymmetric parts independently. Theoretically, the Ott-Antonsen method, the linear stability analysis, and the characteristic method have been respectively carried out to obtain analytical insights. Together with numerical simulations, our study presented the following main results. First, we obtained the low-dimensional description of the symmetric dynamics, and various regimes are predicted in the phase diagram, including the double-center, the single-center, the center-synchrony coexistence, and the synchrony regime. Second, all the steady states and the boundary curves have been obtained analytically both in terms of the Ott-Antonsen ansatz and the linear stability analysis. Third, we proved that in the marginal regime, the stationary symmetry distribution is only neutrally stable, where all the infinitely many eigenvalues are purely imaginary. Finally, the characteristic method has been adopted to obtain the transient dynamics *r*_1_ whose evolution strongly depends on the initial values. Furthermore, for the general case of initial condition, the symmetry dynamics are governed by the Möbius transformation and numerical experiment suggested that three-dimensional invariant manifolds contain neutrally stable chaos in the marginal regime. This work provided a complete framework to deal with the high-order coupling phase oscillators model, and the obtained results will enhance our understandings of the dynamical properties of coupled phase oscillator systems with more higher-order harmonic coupling terms.

## Additional Information

**How to cite this article**: Xu, C. *et al*. Collective dynamics of identical phase oscillators with high-order coupling. *Sci. Rep.* **6**, 31133; doi: 10.1038/srep31133 (2016).

## Supplementary Material

Supplementary Information
