# Supplementary material for: Collective dynamics of identical phase oscillators with high-order coupling
Source: Sci Rep. 2016 Aug 5;6:31133. doi: 10.1038/srep31133 (PMC4974564; doi:10.1038/srep31133)
Supplement: Supplementary Information [file srep31133-s1.pdf]

# Supplementary Material.Collective dynamics of identical phase oscillators with high-order coupling

Can Xu,[1,2,3] Hairong Xiang,[2,3] Jian Gao,[2,3] and Zhigang Zheng[1,*]

[1]*Institute of Systems Science and College of Information Science and Engineering, Huaqiao University, Xiamen 361021, China*
[2]*Department of Physics, Beijing Normal University, Beijing 100875, China*
[3]*Beijing-Hong Kong-Singapore Joint Center for Nonlinear and Complex Systems (Beijing), Beijing Normal University, Beijing 100875, China*
(Dated: April 19, 2016)

The continuity equation for the distribution function $\rho(\varphi, t)$ is

$$\frac{\partial \rho}{\partial t} + \frac{\partial}{\partial \varphi}\left[(\bar{\omega} - 2\lambda \sin\varphi)\rho(\varphi, t)\right] = 0, \tag{1}$$

the stationary distribution is Eq. (20) (in the main text). Linearizing the continuity equation (1) around $\rho_s(\varphi)$, we can write

$$\frac{\partial}{\partial t}(\delta\rho) = \hat{L} \cdot \delta\rho(\varphi, t), \tag{2}$$

where the operator $\hat{L}$ is defined by

$$\hat{L}\,\delta\rho(\varphi, t) = -\frac{\partial}{\partial \varphi}\left[(\bar{\omega} - 2\lambda \sin\varphi)\delta\rho(\varphi, t)\right] - \frac{\partial \rho_s}{\partial \varphi}\int_0^{2\pi} d\varphi'\, \sigma \sin\varphi' \delta\rho(\varphi', t). \tag{3}$$

It is convenient to solve the eigenproblem for the operator $\hat{L}$ in the terms of the basis functions $e^{2\pi i m G(\varphi)}$, where

$$G(\varphi) = \int_0^{\varphi} d\varphi'\, \rho_s(\varphi'). \tag{4}$$

Expanding $\delta\rho(\varphi, t)$ in terms of the basis (Eq. (26) in the main text) and substituting in Eq. (2) one finds that

$$\frac{da_n}{dt} = -2\pi i n C \cdot a_n + b_n \sum_{m=-\infty}^{\infty} f_m\, a_m(t), \tag{5}$$

where

$$b_n = \int_0^{2\pi} d\varphi\, \frac{d\rho_s}{d\varphi}\, e^{-2\pi i n G(\varphi)}, \tag{6}$$

and

$$f_m = -\int_0^{2\pi} d\varphi\, \sigma \sin\varphi\, \rho_s(\varphi) e^{2\pi i m G(\varphi)}. \tag{7}$$

The eigenvalues of $\hat{L}$ are found by diagonalizing the matrix

$$N_{nm} = -2\pi i n C \cdot \delta_{mn} + b_n\, f_m, \tag{8}$$

*Electronic address: zgzheng@hqu.edu.cn

following the same produce in the Ref. [33] in the main text, one obtains

$$b_n = \frac{n\lambda}{\sqrt{\bar{\omega}^2 - 4\lambda^2}} (e^{-2\pi i \bar{G}} \delta_{n,1} - e^{2\pi i \bar{G}} \delta_{n,-1}), \tag{9}$$

where

$$\bar{G} = \frac{1}{2\pi} \arctan \frac{2\lambda}{\sqrt{\bar{\omega}^2 - 4\lambda^2}}. \tag{10}$$

It should be pointed out that in the following part we pay our attention for the case $\bar{\omega} > 0$, while $\bar{\omega} < 0$ the same analysis could be performed. Since $b_n \equiv 0$ for $|n| \neq 1$ and hence the matrix $N$ has corresponding eigenvalues

$$\delta_\ell = -2\pi i \ell \cdot C, \qquad |\ell| \neq 1. \tag{11}$$

In addition to these eigenvalues, there are two extra eigenvalues $\delta_{\pm 1}$, which could be calculated by diagonalizing the two dimensional submatrix

$$\begin{pmatrix} -2\pi i C + b_1 f_1 & b_1 f_{-1} \\ b_{-1} f_1 & 2\pi i C + b_{-1} f_{-1} \end{pmatrix}, \tag{12}$$

after some calculations (Ref.[33] in the main text) one obtains

$$b_1 = \frac{\lambda}{\sqrt{\bar{\omega}^2 - 4\lambda^2}} \left( \frac{\sqrt{\bar{\omega}^2 - 4\lambda^2}}{\bar{\omega}} - i \frac{2\lambda}{\bar{\omega}} \right), \tag{13}$$

and

$$f_1 = -2i\sigma \left( \frac{\sqrt{\bar{\omega}^2 - 4\lambda^2}}{\bar{\omega}} + i \frac{2\lambda}{\bar{\omega}} \right) \frac{\sqrt{\bar{\omega}^2 - 4\lambda^2}}{4\lambda^2} (\bar{\omega} - \sqrt{\bar{\omega}^2 - 4\lambda^2}), \tag{14}$$

and also $b_{-1} = b_1^*$, $f_{-1} = f_1^*$. Substituting these expressions into the submatrix, we find that the extra eigenvalues just correspond to the eigenvalues in the Ott-Antonsen manifold as we discuss the stability of the splay-state in the main text. Further analysis suggests that the two eigenvalues are at most imaginary while the stationary distribution is neutral stable to perturbation in all the directions.
